# Fully Anonymized Digital Health Data Acquisition in a Research Partnership Using a Blinded Deidentification Proxy in the HerzFit App: Implementation Study

**DOI:** 10.2196/77983

**Published:** 2026-05-07

**Authors:** Lara Marie Reimer, Leon Nissen, Fabian Starnecker, Olga Stepanova, Florent Dufour, Ruth Ney, Sinann Al Najem, Heribert Schunkert, Stephan M Jonas

**Affiliations:** 1 Institute for Digital Medicine University Hospital Bonn University of Bonn Bonn, North Rhine-Westphalia Germany; 2 School for Computation, Information and Technology Technical University of Munich Garching bei München Germany; 3 Department of Cardiology German Heart Centre Munich University Hospital of the Technical University of Munich Munich Germany; 4 Partner Site Munich Heart Alliance German Centre for Cardiovascular Research Berlin Germany; 5 ByteLaw Attorneys Frankfurt am Main Germany; 6 Leibniz Supercomputing Centre Garching bei München Germany; 7 Deutsche Herzstiftung Frankfurt am Main Germany

**Keywords:** cardiovascular disease, data anonymization, digital health, GDPR, mHealth, primary prevention

## Abstract

**Background:**

The European General Data Protection Regulation (GDPR) strictly regulates the processing of personal and health-related data, posing challenges for digital health research, especially when data are collected using participants’ own devices. Although scientific data can theoretically be anonymized, standard internet communication protocols inevitably expose transmission metadata, preventing true anonymization. Existing solutions, including virtual private networks, reverse proxies, and trust centers, improve confidentiality but do not technically or legally enable fully anonymized data collection. Consequently, large-scale digital health research often requires extensive organizational measures, complex consent procedures, and high regulatory overhead.

**Objective:**

This study aimed to develop a GDPR-compliant concept for fully anonymized scientific data collection, ensuring that no entity has simultaneous access to identifying information and donated data. We also implemented and evaluated this concept in a real-world public-private partnership.

**Methods:**

We designed a data donation architecture based on a blinded deidentification proxy that decouples identifying transmission metadata from encrypted user data at the time of donation. The concept combines symmetric (Advanced Encryption Standard-128 in Cipher Block Chaining) and asymmetric (Rivest-Shamir-Adleman with Optimal Asymmetric Encryption Padding) encryption, enabling end-to-end encrypted and anonymized data transfer without persistent identifiers. The system was integrated into the HerzFit app, a mobile lifestyle coach for cardiovascular disease prevention available in German-speaking countries, and evaluated for adoption, technical feasibility, and performance. Performance overhead was assessed using round-trip time benchmarks. Duplicate donations were identified and merged to estimate unique data donors.

**Results:**

The solution was integrated and tested in the HerzFit app with more than 200,000 downloads between April 2022 and December 2025. Since the introduction of the data donation feature, more than 13,000 donations have been received, translating to more than 9000 individual users contributing anonymized datasets. Proxy-based transmission resulted in an average round-trip time of 143 ms, compared to 58 ms for direct transfer, representing a modest overhead while maintaining usability. The operator of the donation database did not gain access to identifying information at any stage, demonstrating full technical anonymization. The approach can be operated reliably at scale with minimal server resources due to the stateless proxy design.

**Conclusions:**

This work introduces a novel system architecture enabling fully anonymized, GDPR-compliant data donation directly from participants’ devices. By decoupling identifying metadata from encrypted health data, the concept minimizes regulatory effort, strengthens privacy protection, and provides a practical framework for large-scale digital health research in research partnerships, for example, between a private company and a research institution. The real-world deployment in HerzFit demonstrates the feasibility, scalability, and scientific utility of this approach. The concept is broadly transferable to other mobile health apps and has the potential to substantially expand ethically and legally compliant data acquisition.

## Introduction

The introduction of the European General Data Protection Regulation (GDPR) [1] has been seen as a potential impediment for researchers dealing with identifying information [2,3]. Especially in areas with a large number of digital data acquisition tools and highly sensitive data (eg, social sciences, psychology, or medicine), the process of data collection and the documentation and regulation thereof has become more complex, for example, by requiring documentation of technical and organizational measures or data processing pipelines. One of the most difficult problems to solve is the lack of anonymized data collection through digital tools, as participants in online studies almost always leave identifying data, such as device identifiers or IP addresses.

In the previously mentioned research areas, data acquired directly from the general population are important resources. Mobile devices are of special interest as they contain several integrated sensors that can deliver different kinds of data, such as motion, sound, image, and ambient light data. These data can provide deeper insights into lifestyle factors and digital biomarkers related to the development of diseases or conditions beyond clinical results [4,5]. Another example is ecological momentary assessments in psychological and social sciences or electronic diaries (eg, for mood, pain, or other symptoms), collecting real-time information on behavior or experiences during a person’s daily activities.

PCs, smartphones, and mobile devices are essential resources, as these can gather a plethora of information in the field, without identifying the user per se. In general, obtaining research data from individuals is challenging as it requires compliance with good scientific or clinical practice, ethics approval, and must be performed in a GDPR-compliant way. This even applies when data are donated, for example, through a broad consent [6-8], as the data still contain personal or identifying information. GDPR-compliant data processing includes secure storage, processing, transfer, and documentation of data use [1]. Most importantly, without specific consent, it is not allowed to share, publish, or repurpose parts of the data, as individuals can be reidentified and retain the right of ownership over the data. One potential solution to share data more securely is trust centers, which offer services to store data containing personal information and add strong pseudonymization (eg, in Biobanks [9-11]). Once pseudonymized, the data can be shared with other researchers. However, it still demands high standards for processing sensitive personal data such as health data [11,12], and the GDPR still applies.

Collecting anonymized data for research purposes poses an interesting alternative solution to the aforementioned problems, as the GDPR does not apply to anonymized data [1]. Thus, collecting truly anonymized data are complex by requiring the removal of any identifying information from the dataset and even from any meta-information, especially in a digital setting where the underlying communication protocols require identifying information of both communication partners, even if the data are anonymized and do not contain any information that might identify the data donor. The data transfer from a user’s device to a central database contains personal information such as the IP address, and thus the data are no longer deemed anonymous. Unlike IPv4, where IP addresses are frequently rotated due to limited availability, IPv6 offers a vast address space, where devices potentially obtain stable, long-lived addresses [13]. This shift raises linkability concerns and has significant implications for user privacy, as it enables easier device tracking and online monitoring, potentially enabling reidentification over a longer period of time [14,15]. This implies that the data have to be handled according to the GDPR and can only be anonymized retrospectively by removing the data transfer information.

Current research on anonymous data collection focuses mainly on data anonymization protocols and establishing secure and private communication channels for data exchange [16-19], but does not target data donations from mobile or other personal devices. Although virtual private networks (VPNs), reverse proxies, and pseudonymization services exist, none of them enable legally compliant anonymized data donation from mobile devices under the GDPR. Existing solutions secure data transmission but do not decouple identifying transmission metadata (eg, IP address) from the research dataset. As GDPR guidance and recent legal analyses emphasize, metadata alone is sufficient to render a dataset personal and thus nonanonymous [20-22]. One commonly used approach to obscure a user’s IP address is a VPN. However, VPN providers necessarily process connection logs, timestamps, and the originating IP, which constitutes personal data and allows theoretical relinkage [22,23]. VPNs, therefore, enhance confidentiality but do not satisfy the GDPR’s anonymization test as defined in Recital 26 [20,21]. Similarly, reverse proxies forward requests but do not eliminate identifiers, as backend systems must still process client IP addresses as part of network-level metadata. This means that the data cannot be considered anonymous in the GDPR sense [24]. Pseudonymization services replace identifiers with codes but must retain a mapping or metadata. Under GDPR, pseudonymized data explicitly remain personal data because the possibility of reidentification persists [22,25,26]. Moreover, reidentification research has repeatedly shown that auxiliary information, such as network metadata, can relink individuals to datasets, even when direct identifiers are removed [27,28]. Hence, digital health data collected via apps remain subject to data protection law unless transmission metadata are removed at the moment of data donation, which is not addressed by existing techniques [29].

The aim of our research was thus to design and implement a system architecture that enables fully anonymized, GDPR-compliant data donations directly from the end users’ mobile devices in a research partnership consisting of at least 2 legally separate entities, for example, in collaborations between private entities such as foundations and research institutions. The implementation in the HerzFit app as an example thereof represents a deployment and early scaling phase of a digital health infrastructure within a real-world public-private partnership in Germany, operating alongside national digital health initiatives.

## Methods

### Overview

To target the problem of anonymized data collection using mobile devices, we propose a concept relying on a blinded trust center that enables an anonymized end-to-end encrypted data donation tunnel. In this manuscript, we present the concept and describe its implementation in the HerzFit app [30], a lifestyle app for promoting cardiovascular health in the German-speaking regions. The foundation of the proposed data donation concept was established through a requirements engineering process focused on privacy and data separation. Additionally, a suitable data transfer scheme was selected, and a detailed design was developed, outlining the entities involved and the sequence of events. The concept was then implemented and evaluated within the HerzFit app. We evaluated our implementation regarding its adoption (number of donors), feasibility (successful donations), and performance (round-trip time [RTT]).

### HerzFit

The HerzFit app is a mobile app for cardiovascular prevention available in the German-speaking area with over 200,000 downloads from April 2022 to December 2025 [31,32]. HerzFit’s main goals are to increase risk awareness in primary and secondary prevention of cardiovascular diseases and to improve individual risk-related lifestyle factors. In parallel, the HerzFit app was designed to serve as a tool for scientific data collection to gain more knowledge on lifestyle factors in cardiovascular disease prevention. As such, it enables scientists to collect and analyze several health parameters such as physical activity, resting heart rate, blood pressure, sleep, and laboratory testing results.

HerzFit was developed as part of the DigiMed Bayern research project by the German Heart Centre Munich and the Technical University of Munich (public partners) and is owned and operated by the German Heart Foundation eV (private partner), representing the stakeholders in the research partnership. It was extended by a data donation module in 2023, which was available to iOS users in August 2023 and to Android users in April 2024. Due to this setup, HerzFit serves as an example for the integration of our proposed concept for enabling fully anonymized data acquisition in a research relationship in the field of digital health research.

### Requirements

The proposed data donation concept is subject to several requirements that defines the framework for its design:

#### Separation of Identifying Information From Donated Data

Two legally separate entities are required: entity 1, which is allowed to capture specific personal or identifying information through consent (although no data processing for purposes other than data transfer is needed), and entity 2, which collects and processes the donated data. The legal separation allows the completely anonymous transfer of the data from the client app to entity 2, while limiting entity 1 to processing only minimal personal data required for operating the client app, namely the users’ IP addresses.

#### Full Data Donations

Only single, full donations of the whole user dataset are possible; otherwise, a pseudonym or identifier of any form must be present for record linkage of partial data donations.

#### Anonymizable Dataset

The dataset must not contain any personal or potentially identifying data, such as Global Positioning System (GPS) data or the hardware fingerprint (MAC address, etc).

#### No Direct Communication Between Donor and Receiver

The data cannot be sent directly from the user’s device, as the transmission would require the exchange of the user’s IP address.

### Data Transfer Schemes

In general, several possibilities exist for transferring scientific data through the internet. They can be divided into 3 main schemes: direct transfer, across a trust center, and using a proxy for the transfer.

**Figure 1:**
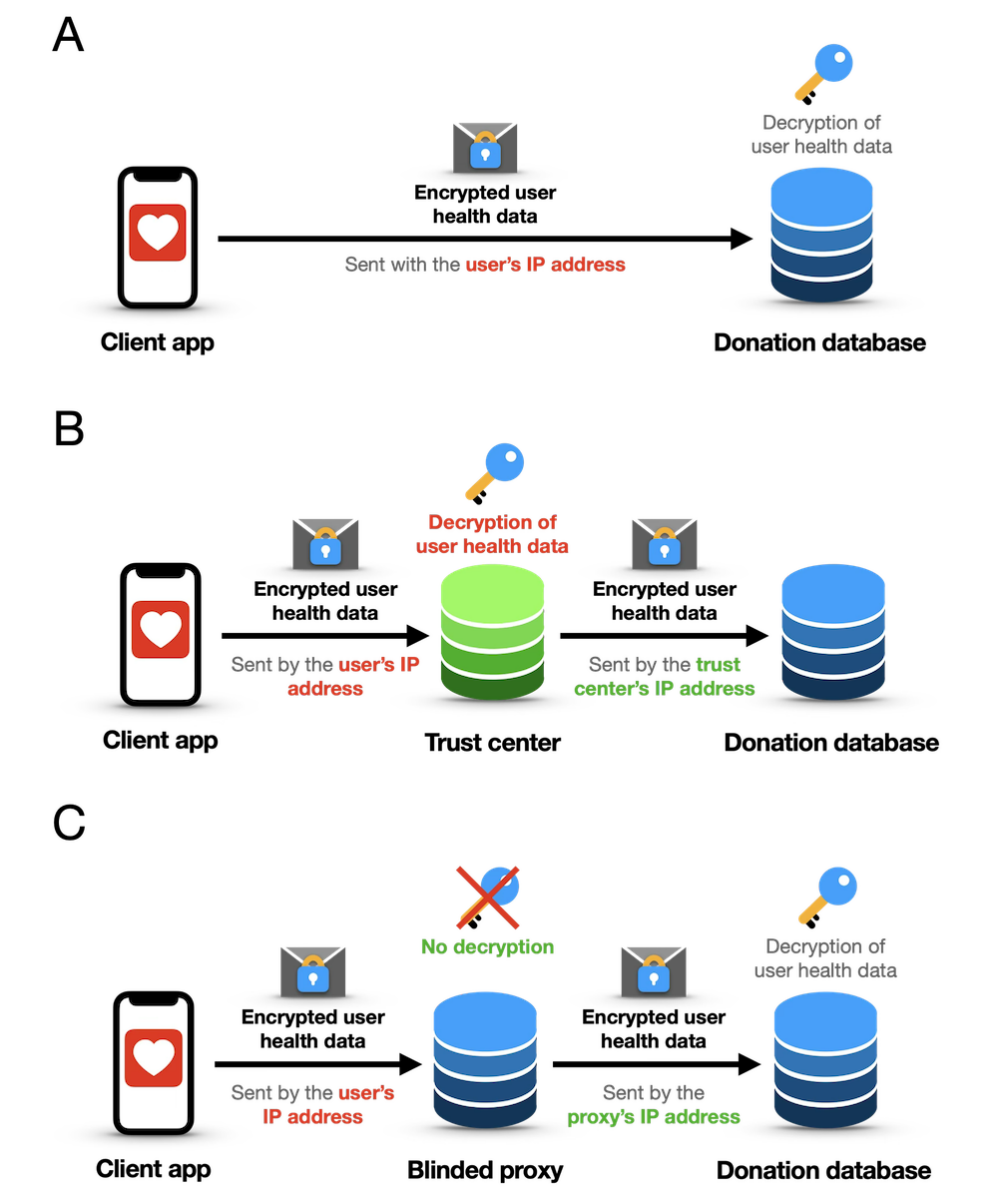
Illustrations of different data transfer schemes from the client app to the donation database. (A) Direct data transfer scheme: while the health data themselves can be anonymized, they are still associated with the user’s IP address, thus linked to identifying information. (B) Trust center data transfer scheme: as the data are transferred directly from the users’ devices to the trust center and further distributed from there, the stored data are strongly pseudonymized. (C) Deidentification/blinded proxy scheme: the blinded proxy forwards the encrypted data using its own IP address, thus enabling a fully anonymous transfer.

Using the direct transfer scheme (Figure 1A), the data are sent directly from the user’s device to the server storing the scientific data. As this transfer has to happen via standardized IPs (ie, TCP/IP), the data are sent from and to the user’s IP address, which must therefore be known by the connected server. Until 2022, the GDPR considered IP addresses as personal information, as, in theory, users could directly be identified through them (GDPR Consideration 30 [1]). In 2022, the European Court of Justice (ECJ) ruled (C-319/22) that alphanumeric codes, including IP addresses, do not constitute personal data as long as no one is able to draw conclusions about the person to whom this IP address belongs from the IP address by reasonable means [33]. However, this interpretation may be challenged by the evolution of IPv6, where Stateless Address Autoconfiguration (SLAAC) can generate long-lived interface identifiers, potentially derived from device MAC addresses, thereby enabling stable, device-specific IP addresses unless privacy extensions are used [14,15,34]. In addition, it is unclear if the IP address, in combination with medical information, is still not considered personal data.

Therefore, direct data transfer always contains personal and identifying information, even though the data themselves might be anonymized.

In contrast, trust centers serve as independent third parties for receiving, storing, managing, and sharing data or personal information for specific purposes. Their main advantage is that they can establish strong pseudonymization of data collected for scientific purposes. In the trust center scheme (Figure 1B), users would send their data to the trust center, which shares these data with researchers under different pseudonyms, thereby reducing the reidentification or recombination risk. As the trust center handles identifying information (at least the IP address), GDPR regulations apply to the trust center for processing the data.

The proxy data transfer scheme or proxy pattern is a widely used architectural pattern to access another software component and to perform operations on incoming/outgoing objects [35]. In the specific case of data transfer, a proxy can be used as a blinded trust center that only forwards information using its own IP address, thus not operating on the transferred object itself, but being used for anonymous data transfer by acting as a deidentification proxy (Figure 1C). By using encryption on the transferred data, the proxy cannot access any (personal) data beyond the IP address, does not handle any sensitive user data, and is subject to less strict regulations. Moreover, it enables the completely anonymous transfer of the user data to researchers. Based on these 2 advantages of the proxy pattern, this data transfer scheme was chosen as the underlying architecture of the proposed data collection concept.

### Concept Design

Based on the described requirements, a concept was designed to enable scientific data collection. It encompasses 3 entities enabling a data flow for deidentified data donations using a blinded proxy.

#### Entities

##### Overview

The concept entails three main entities: (1) the mobile client app, which is responsible for acquiring informed consent and data collection, (2) a deidentification proxy owned and operated by legal entity 1 acting as a data trust agency, and (3) the donation database, owned and operated by legal entity 2. The client app and the deidentification proxy are operated by entity 1, while the donation database is operated by entity 2.

##### Client App

The client app can be any mobile app collecting data of interest for research. The app should provide informed consent that includes details on which data are being stored and the purpose of the data donation. After the informed consent is given, the app can start data collection. Once the desired amount of data have been collected, the app removes any personally identifiable parts from the data to achieve anonymization, encrypts the data using entity 2’s public key, and sends it to the proxy server. By implementing data donations according to the proposed concept, no additional GDPR duties arise for the app operators (apart from handling the users’ IP addresses, which is commonly done anyway).

##### Deidentification Proxy

The deidentification proxy decouples the encrypted health data from the IP address sent from the client app. It forwards the data to the donation database using its own IP address and notifies the client app about the operation’s success. For this purpose, the deidentification proxy is owned and operated by legal entity 1, which has permission to process personal data and acts as a data trust agency for the forwarding process. As the health data are encrypted and cannot be decrypted by the deidentification proxy, the deidentification proxy only handles IP addresses as personal data and is thus subject to lower data protection regulations.

##### Donation Database

The donation database is owned and operated by legal entity 2. It is responsible for storing the data and responding to the successful receipt of the data. The data can then be decrypted by legal entity 2’s private key. All data that reach the donation database are fully anonymized and encrypted. The data are sent from the deidentification proxy’s IP address. These 2 conditions imply that the GDPR regulations do not apply anymore, as no personal information is available.

#### Flow of Events

**Figure 2:**
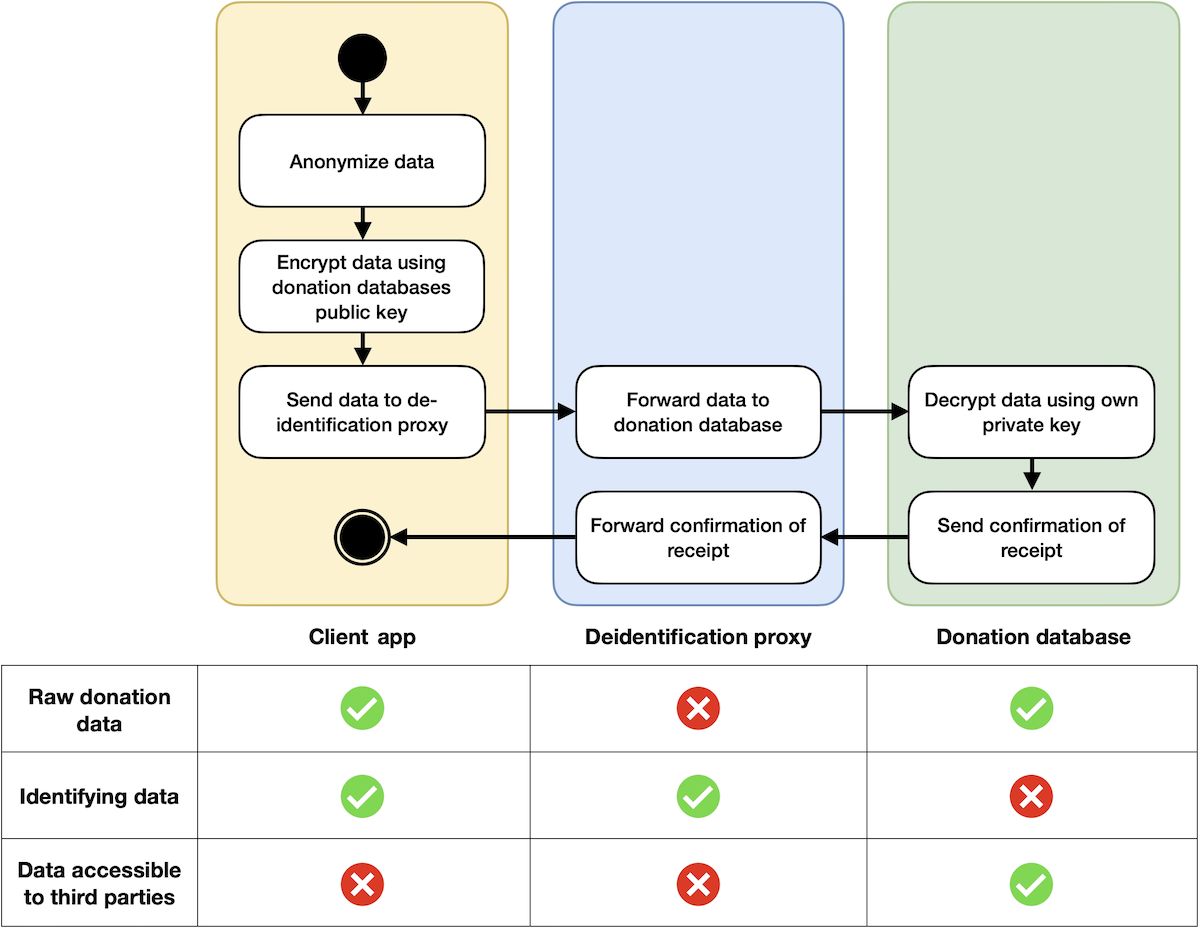
Flow of events of a data donation between the 3 entities: client app, deidentification proxy, and donation database. Through neither of the 3 entities, any third party can get access to the data donor’s data and identifying information at the same time, enabling a fully anonymized data donation.

The event flow starts with the client app anonymizing the relevant data collected previously (Figure 2). The collected data are subjected to a 2-tier encryption process that combines symmetric and asymmetric cryptography. Symmetric encryption is used to protect the collected data itself. It relies on a single secret key for both encryption and decryption and is computationally efficient, making it well-suited for encrypting large volumes of data such as the datasets considered in this work. The main challenge of symmetric encryption lies in the secure distribution of the secret key. To address this, asymmetric encryption is used to protect the symmetric key: a key encrypted with a public key can only be decrypted using the corresponding private key. This enables secure key exchange without requiring a preshared secret. It is therefore common to use symmetric encryption to encrypt the data, while asymmetric encryption is used to safely transfer the key or passcode [36]. More details on the cryptography are provided in the “Validity of the Cryptographic Process” section.

Next, the client app sends the encrypted symmetric key and encrypted health data to the deidentification proxy. Upon receipt of the donation, the deidentification proxy forwards the encrypted health data and encrypted symmetric key to the donation database. The deidentification proxy then awaits a response from the donation database, which it relays back to the client to inform them about the successful transmission or any occurring error. Regardless of the response from the donation database, the deidentification proxy ensures that the temporarily stored data are deleted after the transaction is completed. In case of any occurring error, it is up to the client app to retry to donate the data later.

The donation database receives the data donation and encryption key and replies with a success or failure message. It stores the encrypted health data on disk, then decrypts the symmetric key using the private asymmetric key and subsequently decrypts the health data using the symmetric key. Successful decryption prompts the donation database to inform the deidentification proxy, which in turn notifies the client app of the successful data donation. If errors occur during the process (eg, decryption does not work due to malformed data or faulty transmission), a corresponding error code is sent as a reply.

At no stage in this process does any party but the donor have access to identifying information (IP address) and the donation data simultaneously. The deidentification proxy decouples the identifying information and donated data. The data donation can be considered fully anonymized (Figure 2).

#### Validity of the Cryptographic Process

We demonstrate that the data transfer solution ensures confidentiality under standard cryptographic assumptions. We use a standard hybrid encryption scheme. The plaintext donation data *m* are efficiently encrypted with a symmetric encryption key *k_S_*, and this symmetric key is securely encapsulated and provided to the donation database after asymmetric encryption using its public key *pk_D_*.

**Figure 3:**
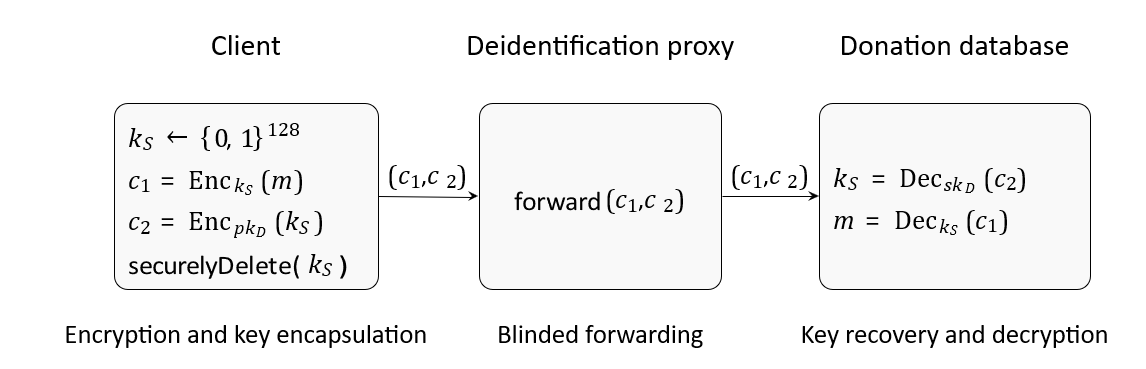
Formalization of the cryptographic process used in the data donation workflow. The client generates a fresh symmetric key *k_S_*, encrypts the clear-text donation data *m* with Advanced Encryption Standard-128 in Cipher Block Chaining, and encrypts *k_S_* with the donation database’s Rivest-Shamir-Adleman–Optimal Asymmetric Encryption Padding public key *pk_D_*. The proxy performs no cryptographic operations and blindly forwards the ciphertexts (*c_1_*,*c_2_*) without the ability to extract any information. The donation database server receives (*c_1_*,*c_2_*) from the proxy IP address and recovers *k_S_* using *sk_D_* and decrypts *m*.

For the symmetric encryption of the donation data, we use Advanced Encryption Standard (AES)-128 in Cipher Block Chaining mode. This construction provides semantic security (Indistinguishability under Chosen-Plaintext Attack) when used with random initialization vectors, ensuring that a passive adversary (including the proxy) cannot distinguish plaintexts or learn information about the encrypted health data [37,38]. For key encapsulation, we use Rivest-Shamir-Adleman (RSA) with Optimal Asymmetric Encryption Padding as standardized in Public-Key Cryptography Standards #1, which provides protection against chosen ciphertext attacks (Indistinguishability under Chosen-Ciphertext Attack) in the random oracle model [36,39]. These standard primitives ensure that neither the proxy nor an active network adversary can derive information about the symmetric key *k_S_* or the plaintext *m*. An adversary controlling the communication channel (including the proxy) cannot derive the symmetric key *k_S_* nor any information about the plaintext *m*. Compromise of the proxy leaks only routing metadata. Because *k_S_* is freshly generated per donation and destroyed after transmission, compromise of one symmetric key does not affect other donations. Only the compromise of *sk_D_* endangers the confidentiality of stored encrypted donations. The cryptographic protocol is formalized in Figure 3.

### Ethical Considerations

**Figure 4:**
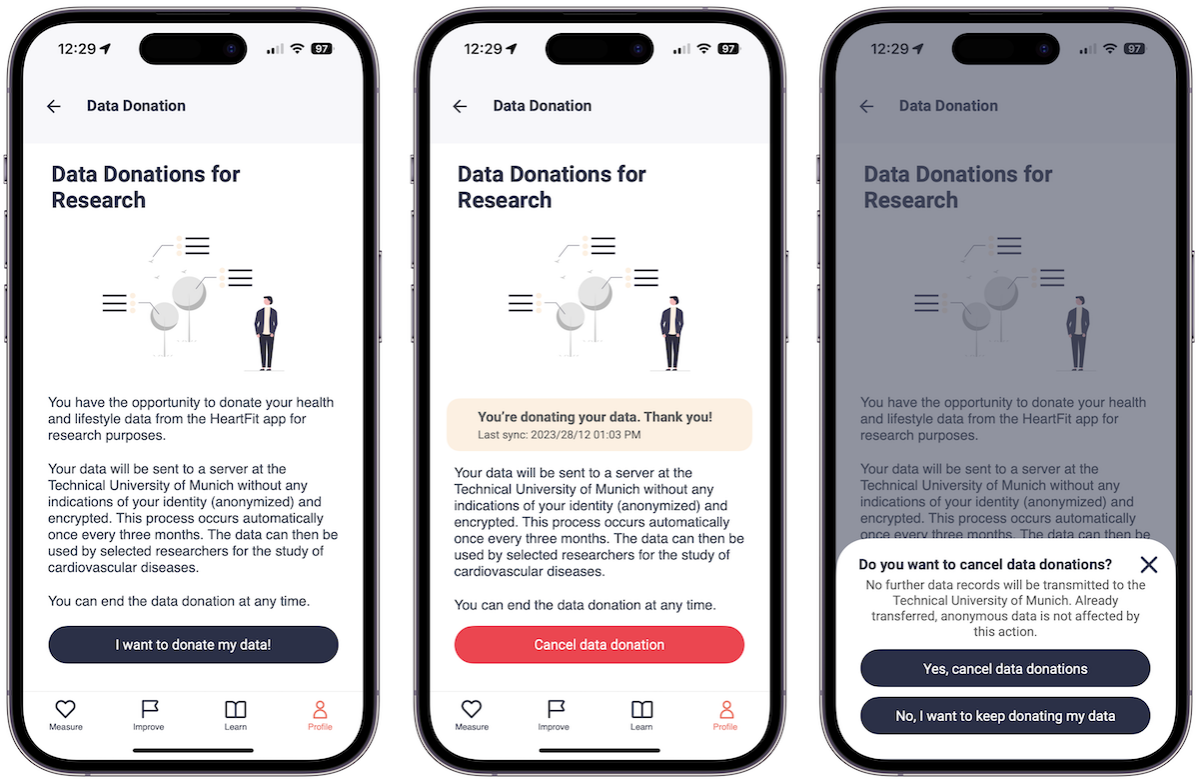
Screenshots of the HerzFit app depicting the process of managing data donations from the data donors’ perspective: informed consent and starting the donations (left), information on the last donation and the possibility of canceling future donations (center), and canceling donations dialogue (right).

The collection, transfer, and analysis of data donated in the HerzFit app were approved by the Ethics Committee of the Technical University of Munich in November 2022 (315/20 S-EB). All data donors gave informed consent through the HerzFit app (Figure 4). Only anonymous data were donated. The data donation concept, in its implementation in the HerzFit app, was developed in close collaboration between the scientific and private stakeholders. It was presented to the data protection officer of the German Heart Foundation as well as the Bavarian Data Protection Commissioner and was approved in March 2023.

## Results

### Overview

In contrast to the established concept of trust centers, our data donation concept introduces a deidentification proxy, which can be operated by the mobile app owner, thus making a separate, third entity for managing the nonanonymous donation data obsolete. The complete decoupling of identifying information and the donation data ensures full anonymization throughout the process. To test and validate the concept, it was integrated into the HerzFit app and complemented by several additional measures, further described within this section. However, the concept is transferable to any app operated by a stakeholder who would like to allow their app users to donate their data for research.

### Implementation of the Data Donation Process in the HerzFit App

The data donation system was integrated into the HerzFit app in 2023. To start the data donations, data donors must give informed consent. HerzFit has, therefore, been extended by additional screens informing users about the data donations, their purpose, and the donation process (Figure 4). For each data donation, all values from HerzFit’s local database, for example, health and questionnaire data, are collected and prepared for transmission.

**Figure 5:**
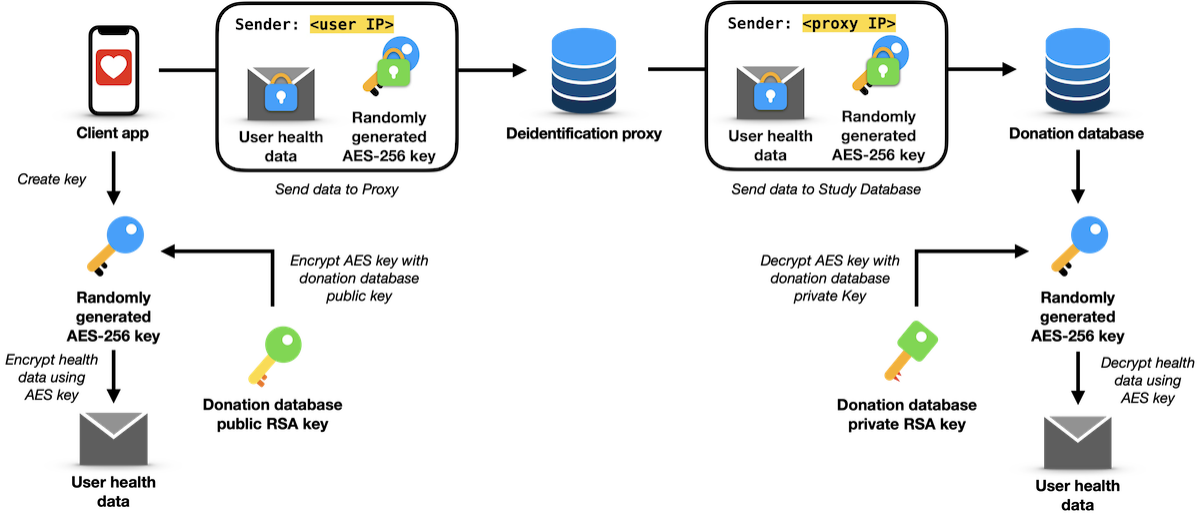
Graphical overview of the process of a data donation. AES: Advanced Encryption Standard; RSA: Rivest-Shamir-Adleman.

To transfer the data securely via the proxy, an end-to-end encryption between the client’s device and the donation database is established (Figure 5). This requires a combination of symmetric (here, AES) and asymmetric (here, RSA) encryption. The RSA key is created using the node:crypto library, and the AES key is generated using react-native-aes-crypto. The data donation is stored in CSV format and then encrypted. The data transfer is performed using the fetch command of the Node JavaScript (Node.js) library. The donation database stores the decrypted CSV files on disk.

After successful transmission, HerzFit keeps a record of the transmission data. HerzFit automatically donates the donor’s complete datasets every 3 months, sending a reminder to the data donors 24 hours before the next donation to allow them to opt out again. If data donors decide to opt out, they will be reminded that no further data will be donated, but their already donated, anonymous data will still remain on the research servers. Anonymized data cannot be deleted, as no direct connection between an individual and the data can be made, and it is therefore not possible to select a dataset for deletion. The data donation does not rely on clinical interoperability standards such as HL7 Fast Healthcare Interoperability Resources, as data donations occur as anonymized batch exports. However, the architecture is payload-agnostic and compatible with standardized data models.

As HerzFit users donate their full dataset every 3 months, the database eventually contains several datasets per donor. Ideally, only the latest donation should be used for further analysis. Datasets are therefore merged, assuming that each subsequent donation will contain a significantly large subset of the previous donation. Using this approach, we identified 8872 potential duplicates in the donations in October 2025, mapped to 4229 individual users, resulting in a total number of 9166 users having donated their data.

### Security Measures in HerzFit

A combination of technical and organizational measures is implemented to ensure the security and privacy of the collected research data. We use strong encryption and access control techniques to secure the collected data.

#### Data Security via Encryption

In addition to the encryption process presented in this paper, our implementation adheres to the confidential computing threat model [40], encrypting data in transit, at rest, and in use. Data in transit are encrypted at both the content and transfer level (HTTPS). Data at rest are encrypted using file system encryption. Data in use are encrypted using a trusted execution environment in the form of an encrypted confidential virtual machine running the donation database engine.

#### Data Security via Strict Access Control

We created a zero-trust architecture in which users, devices, and apps are always authenticated with minimal privileges granted. Devices that communicate with each other systematically verify each other’s identity with pinned certificates and Internet Protocol Security, preventing person-in-the-middle attacks. The database server is secured by a firewall that denies unauthorized connections.

### Overhead, Scalability, Budget, Sustainability, and Generalizability

Enabling data donations at scale also requires considerations regarding potential overhead costs due to the slightly more complex architecture, the possibility to scale the approach, as well as the generalizability to other settings. Our system requires the operation of one additional server: the donation proxy, introducing additional costs for operation as well as additional latency in the data transfer. In internal tests, sending the data through the proxy took an average RTT of 143*.*3 ms, compared to an average RTT of 58*.*8 ms when transferring data directly. While this resembles a nearly tripling of RTT, the latency still remains in reasonable areas. Furthermore, the donation proxy can be run in a stateless, on-demand configuration, only requiring usage capacity when it is actually needed, keeping potential server costs at a minimum. In HerzFit, minimal server configurations are sufficient, highlighting the insignificance of additional costs caused by the donation proxy. The implementation in HerzFit thus required only minimal budget beyond existing infrastructure. Furthermore, sustainability is ensured through integration into the ongoing operation of the HerzFit app, ownership by a nonprofit foundation, and minimal recurring infrastructure costs. Generalizability to other systems is given, since the system is payload-agnostic. Furthermore, since HerzFit’s data are only donated every 3 months, depending on the individual user’s first donation, the donation system is barely at its capacity. The possibility of running the donation proxy in a stateless configuration further highlights its possibilities for horizontal scaling and adds load-balancing if necessary. Requirements for transfer to other setups are thus rather given by the legal setup, including considerations on data deidentification (see “Data Deidentification” section) and the setup in a research partnership.

### Lessons Learned

The implementation of fully anonymized data donation in a real-world digital health app revealed several important lessons. First, the absence of persistent identifiers, while essential for achieving anonymization, complicates downstream data management. Identifying and merging duplicate donations is an error-prone process, as deduplication can only rely on heuristic and statistical approaches rather than deterministic identifiers. Consequently, duplicates can be reduced but not fully eliminated. Second, anonymization appears to increase user trust and willingness to donate data, as reflected by sustained adoption in the HerzFit app. However, anonymity introduces operational constraints, such as the inability to selectively withdraw previously donated data or to recontact donors, which complicates longitudinal data collection and quality control. Third, developing a robust and compliant data donation system required a deep understanding of data protection law, particularly the distinction between anonymization and pseudonymization under the GDPR. Early and continuous involvement of legal expertise was therefore essential.

## Discussion

### Summary and Key Findings

The proposed data donation concept provides a novel technical and organizational framework for enabling fully anonymized, GDPR-compliant data donations directly from users’ mobile devices in a research partnership, thus fully achieving this research’s objective. By introducing a deidentification proxy that decouples transmission metadata from encrypted health data, the system overcomes limitations of existing approaches such as VPNs, reverse proxies, and trust centers, all of which fail to meet the GDPR’s anonymization requirements due to the unavoidable processing of personal identifiers. Our implementation within the HerzFit app demonstrates the feasibility of this architecture at scale: among more than 200,000 app downloads, 9166 users donated data, showing substantial uptake in a real-world public-private partnership setting. The system operated reliably with only moderate performance overhead, confirming that anonymization can be achieved without compromising usability.

In addition to meeting strict legal and technical criteria for anonymization, the concept offers meaningful real-world implications. It reduces the regulatory burden for data controllers by ensuring that no entity ever possesses both identifying information and research data, thereby exempting anonymized donations from the GDPR after transmission. This facilitates large-scale, ethically sound digital health research while preserving user autonomy and privacy. The approach is generalizable to other mobile health apps, provided that deidentifiable data are collected, and enables new forms of collaboration between app operators and research institutions. Together, these contributions position the proposed architecture as a significant advancement for secure, scalable, and privacy-preserving data acquisition in digital medicine.

### Fulfillment of Legal Requirements

#### Fulfillment of Anonymization

In the context of collecting personal data for research purposes, the problem of handling data appropriately arises repeatedly in light of the GDPR and other related laws. One way to avoid the applicability of the GDPR is to anonymize personal data. In accordance with the principles of data protection, the GDPR only applies to information relating to an identified or identifiable natural person. Data that has no personal reference or has been anonymized to a sufficient extent so that the data subject cannot or can no longer be identified does not fall within the scope of the GDPR (Recital 26 GDPR [1]). The processing of such anonymized data, including for statistical and research purposes, is therefore not subject to the strict requirements of GDPR and can be used freely. When assessing whether a natural person is identifiable, Recital 26 states that all means that could reasonably be expected to be used by the controller or another person to identify the natural person directly or indirectly, such as singling out, must be considered. When deciding whether means could reasonably be expected to be used to identify the natural person, all objective factors must be considered, such as the cost of identification and the time required, as well as the technology available at the time of processing and technological developments. Since the proposed data donation concept is based on a deidentification proxy, in which the donor’s data are anonymized and only encrypted data are forwarded to the donation database via the deidentification proxy, it is extremely unlikely that third parties will gain knowledge of it in accordance with the requirements of Recital 26 set out above. As the sole identifying information, the IP address of the data donor is used only for the transmission between the user and the proxy server. At no other point in time is identifying information used or transmitted. On November 9, 2023, the ECJ ruled (C-319/22) that alphanumeric codes, including IP addresses, do not constitute personal data as long as no one can draw conclusions about the person to whom this IP address belongs from the IP address by reasonable means [33]. This is an important relativization of the prior Breyer-decision of the ECJ (C-582/14) after which IP addresses were broadly understood as personal data [41]. Yet, given the increased use of IPv6, this decision might be reconsidered. HerzFit is able to anonymize all personal data and even shield the IP address from the donation database by using the deidentification proxy method. By using this method, the data used are therefore considered completely anonymized. Even when considering the IP address personal data, it is processed under the consent between the user and the operator of the anonymization proxy (here, the German Heart Foundation).

#### Difference Between Trust Center—Deidentification Proxy

When using a trust center architecture to transfer data, a third party, the so-called trust center, is placed between the sender and the recipient. The concept of a trust center works as follows: instead of transmitting the relevant information directly from the sender to the recipient, it is first transmitted to the trust center. The trust center checks the transmitted data for accuracy and only then sends it to the recipient. However, such a procedure has the problem that the data are not encrypted before it is transmitted to the trust center, and therefore third parties gain knowledge of the data, which means that the data are not anonymized and therefore fall within the scope of the GDPR. Instead of forwarding the donated data via trust center, the HerzFit app uses the described data donation process via a deidentification proxy. The collected data are encrypted and anonymized on the sender’s device and then sent to the recipient without being forwarded to a trust center or other third party. The recipient is then able to decrypt and use these data. Compared to the use of a trust center architecture, the use of this method can guarantee complete anonymization of data.

#### Requirements for GDPR-Compliant Data Processing

To understand the benefits of the described data donation concept, it is important to examine the basic provisions of the GDPR and understand its extensive scope of application and requirements for data protection–compliant processing. According to Article 3 GDPR, the regulation applies to all personal data processed in the context of the activities of an establishment of a controller (or a processor) in the European Union (EU). Processing can also occur outside the EU, as the regulation applies to non-EU controllers processing EU data subjects’ personal data when offering goods or services in the EU or monitoring their behavior. In addition to the far-reaching scope of application, the requirements for the processing itself are also extensive and therefore entail a comprehensive data protection concept. In accordance with the principle of purpose limitation and data minimization (Article 5 para 1 GDPR), data may not simply be collected without a reason. To this end, Article 6 GDPR stipulates a number of conditions for lawful processing, which must be met alternatively. Within the scope of the data donation activities, the lawfulness of the processing is based on the data subject’s consent (Article 6 para 1 lit a, Article 9 para 2 lit a GDPR). A number of requirements must be met to obtain a valid consent. First, the declaration of consent must have been given unambiguously. Second, the declaration must have been made voluntarily, and there must be no clear imbalance between the controller and the data subject. Although the declaration of consent does not require a specific form, it must be made in an informed manner. This means that the declaration must be clear and comprehensible and must state who the controller is and for what purposes the data are being processed. The data subject must also be informed of their right of withdrawal in the declaration. Particularly for collecting health data, as is the case with HerzFit, even stricter requirements for valid consent apply. According to Article 9 GDPR, consent must be given expressly; implied consent is therefore excluded. It should also be noted that the controller bears the burden of proof of validly obtaining consent (Article 5 para 2 GDPR). If the consent does not meet the requirements described, it is invalid, and the processing is unlawful in its entirety. This can have far-reaching consequences, such as the imposition of fines in accordance with Article 83 para 5 lit a GDPR or claims for damages by the data subject according to Article 82 GDPR. As data controllers must meet a large number of requirements for data protection-compliant processing and the scope of the GDPR is broad, this creates an enormous amount of work for data controllers, which is often associated with a large investment of resources such as money, time, and personnel. This can be an enormous burden, especially for small and medium-sized companies. The data donation concept avoids these high requirements by completely anonymizing the data used and, therefore, not falling within the scope of the GDPR. This benefits both the data controller and the data subject, as the data can be used securely [1].

### Data Deidentification

The fulfillment of anonymization depends on the transferred data. If the data contains data types that are still identifiable, either because they are not anonymizable to a sufficient extent per se (eg, genetic data, GPS positions, electrocardiogram recordings, etc), or just not sufficiently anonymized, the anonymity of the transmission concept is rendered irrelevant. In this case, the GDPR regulations would still apply to the organization receiving data donations since the data would be considered identifiable.

Data deidentification is an important yet complex topic and is thus out of scope in this research, which focuses primarily on outlining a system-based approach for anonymous data transfer. However, we want to highlight that respective measures need to be taken by the client app provider to ensure that only completely deidentified data are transferred for data donations.

In the case of HerzFit, only deidentified data are transferred. In general, HerzFit does not collect and store any personal or identifiable data, since no accounts are created and all data collected are not considered biometric. No data regarding names or other personal identifiers is stored in or transmitted from the app. HerzFit does not collect biometric or hardly anonymizable data such as electrocardiogram or GPS data, and thus neither donates these data types.

### Security Analysis and Threat Mitigation

#### Overview

Our threat model assumes a hostile environment where network components may be compromised. We analyze the security of the proposed architecture with respect to the CIA triad (confidentiality, integrity, and availability). We additionally discuss malicious clients and privacy risks. We use notations introduced in the “Validity of the Cryptographic Process” section and Figure 3.

#### Trust Model

The donation database is the only fully trusted entity. It holds the private decryption key (*sk_D_*). The client app is trusted to collect data, but is untrusted regarding data coherence. The deidentification proxy is semitrusted; it is trusted to mask IP addresses but is considered an adversary regarding data confidentiality and integrity.

#### Attacks Exploiting the Deidentification Proxy

We assume an adversary may compromise the proxy to target the confidentiality, integrity, or availability of the system.

#### Confidentiality

The proxy is designed to be oblivious to the payload content. It forwards the encrypted payload (*c*_1_*,c*_2_) but possesses neither the symmetric key *k_S_* nor the database’s private key *sk_D_*. The semantic security of the encryption scheme ensures that sensitive medical data confidentiality is preserved. A compromised proxy sees only ciphertext and IP addresses. Sensitive medical data confidentiality is preserved. Since *pk_D_* is not rotated in the current implementation, slight risks emerge in terms of forward secrecy. However, since *sk_D_* is securely stored on the donation database server, which is protected through several measures, compromising *sk_D_* is unlikely.

#### Integrity

The AES in Cipher Block Chaining mode does not inherently guarantee the integrity of the data and is malleable. In this design, integrity is primarily provided by Transport Layer Security (TLS) to ensure end-to-end cryptographic integrity at the payload layer. The donation database detects verification failures during the decryption process and discards the payload, ensuring that only data with verified integrity is processed.

#### Availability

The proxy is publicly accessible and thus susceptible to Denial of Service attacks. While the system does not include specific defenses against volumetric Distributed Denial of Service attacks, the proxy’s stateless design prevents asymmetric resource exhaustion attacks (eg, memory depletion). Crucially, the data donation process is asynchronous and delay-tolerant; temporary unavailability does not result in data loss, as the client app queues the donation for a later retry.

#### Malicious Clients and Data Coherence

Anonymity prevents the permanent banning of malicious users (client app). A malicious client could attempt to inject garbage or plausible but false data (poisoning) by tampering with the app’s normal operations. *Syntactic validation* occurs immediately after decryption; payloads failing CSV schema validation are rejected. *Semantic coherence* is ensured during the data analysis phase. We apply plausibility filters (eg, rejecting physiological impossibilities like a resting heart rate of >250 bpm) and statistical outlier detection to identify and exclude incoherent data points.

#### Network Interception and Spoofing

An adversary may attempt to intercept traffic before it reaches the proxy (person-in-the-middle) or impersonate the legitimate proxy server entirely (spoofing).

#### Interception

Communication between the client and proxy is secured via standard TLS. The client authenticates the proxy by validating its certificate against trusted root certificate authorities. This prevents person-in-the-middle attacks, as an adversary would require a valid certificate signed by a trusted certificate authority to intercept the connection.

#### Spoofing

If a client is redirected to a spoofed proxy (eg, via Domain Name System poisoning), the inner layer of encryption protects the data. The spoofed proxy receives (*c*_1_*,c*_2_) but cannot decrypt it without *sk_D_*. The attack is limited to a denial of service (effectively dropping the donation).

#### Privacy Risks

Even with secure transmission, privacy risks exist regarding the data payload and network metadata.

#### Reidentification Attacks

An attacker may attempt to link the anonymous dataset to specific individuals [27,42]. If successful, this would demonstrate that the data constitutes personal data, falling under the GDPR scope [43]. To mitigate this, the donation database is strictly isolated and accessible only to designated researchers. Furthermore, the dataset is minimized to common physiological parameters, excluding unique identifiers or high-entropy attributes that facilitate linkage.

#### IPv6 and Long-Term Linkability

While IPv4 addresses are frequently rotated, IPv6 [13] introduces privacy challenges via SLAAC. Traditional SLAAC implementations (Extended Unique Identifier-64) generate static interface identifiers derived from the device’s MAC address [34]. This could theoretically allow a compromised proxy to track a user’s device over its lifetime. To mitigate this, we rely on modern mobile operating systems implementing privacy extensions (Request for Comments [RFC] 4941, updated by RFC 8981 [15]), which generate “privacy extensions” (temporary, randomized IP addresses) for outgoing connections, thereby disrupting long-term linkability at the network layer.

### Limitations

The data donation concept presented in this research is limited by the specific conditions that need to be fulfilled to be able to apply it, most importantly, the fact that 2 separate legal parties are needed to establish the anonymization data flow. Without a second entity, the requirement of anonymity cannot be accomplished due to the transmission of metadata connected to any transmission via the internet.

Moreover, the concept is further limited by the data collected by the client app, depending on the anonymizability of the data collected as described in the “Data Deidentification” section. Even if anonymization is fulfilled from a technical perspective, it cannot be achieved if the transmitted data contains identifying information. The concept does not include any mechanism for data deidentification beyond the data transfer. If the collected data contains identifying information (eg, genetic data, GPS positions, etc) that is either not anonymizable to a sufficient extent or just not sufficiently anonymized (eg, it still contains identifying metadata), the GDPR regulations still apply to the organization receiving data donations, and the transferred data would not be anonymized.

### Conclusion

In this work, we presented that implementing fully anonymized data donation for digital health research within a research partnership consisting of at least 2 legally separate entities is technically and organizationally feasible when identifying transmission metadata are decoupled from encrypted research data at the moment of transfer. By leveraging a deidentification proxy, we demonstrated how sensitive health data can be collected and transferred without exposing identifying information, thus ensuring compliance with GDPR while maintaining scientific use. The implementation of this concept in the HerzFit app has validated its feasibility, with over 9166 users contributing anonymized health data since the introduction of the data donation feature. The approach establishes a practical pathway for ethically and legally compliant data acquisition in digital health, reducing regulatory burden, strengthening user privacy, and enabling long-term collaborations between app operators and research institutions.

Compared to traditional trust center architectures, our approach minimizes regulatory burdens and enhances privacy by decoupling the transmission of personal identifiers from the donated data. This makes anonymized data collection more accessible and scalable for future research. Nevertheless, legal and technological developments, such as the increasing adoption of IPv6, may require ongoing adaptations to maintain compliance and security. Future work should focus on refining the framework for broader adoption and assessing its long-term impact on digital health research. Furthermore, the system’s current threat model could be further refined and improved, for example, by ensuring forward secrecy and enabling certificate pinning.

The presented architecture offers a foundation for future applications and can be transferred to other mobile health contexts where deidentified data are collected. By bridging technical design with regulatory requirements, this work provides a scalable and generalizable framework that can help unlock broader, population-level insights in digital medicine.

## Data Availability

The datasets generated or analyzed during this study are available from the corresponding author on reasonable request.

## Abbreviations

AES: Advanced Encryption Standard
ECJ: European Court of Justice
EU: European Union
GDPR: General Data Protection Regulation
GPS: Global Positioning System
RFC: Request for Comments
RSA: Rivest-Shamir-Adleman
RTT: round-trip time
SLAAC: Stateless Address Autoconfiguration
TLS: Transport Layer Security
VPN: virtual private network
